# Crisis Text Line and Loris.ai Controversy Highlights the Complexity of Informed Consent on the Internet and Data-Sharing Ethics for Machine Learning and Research

**DOI:** 10.2196/67878

**Published:** 2025-01-22

**Authors:** Gunther Eysenbach

**Affiliations:** 1 JMIR Publications Toronto, ON Canada

**Keywords:** data ethics, data sharing, informed consent, disclosure, conflict of interest, transparency, trust

In January 2019, the *Journal of Medical Internet Research* published a viewpoint paper by Anthony Pisani and colleagues [1] related to a noncommercial data-sharing program for academic researchers offered by Crisis Text Line, a not-for-profit technology organization that provides a free 24-7 text line for people in crisis in the United States. Crisis Text Line has been described as a globally prominent online mental health support resource, where people in crisis (eg, with suicidal ideation) can exchange text messages with trained volunteer counselors. As is the case for many internet companies, these digital exchanges are a treasure trove for machine learning, quality improvement, education, and research.

Nancy Lublin, cofounder and former chief executive officer (CEO) of Crisis Text Line (as well as Loris.ai), gave an example in her 2015 TED talk on the internal use of these data [2]:

We know that if you text the words “numbs” and “sleeve,” there's a 99 percent match for cutting. We know that if you text in the words “mg” and “rubber band,” there's a 99 percent match for substance abuse. And we know that if you text in “sex,” “oral” and “Mormon,” you're questioning if you're gay. Now that's interesting information that a counselor could figure out but that algorithm in our hands means that an automatic pop-up says, “99 percent match for cutting -- try asking one of these questions” to prompt the counselor. Or “99 percent match for substance abuse, here are three drug clinics near the texter.” It makes us more accurate.

Former Crisis Text Line board member, Danah Boyd, recounts her motivation behind opening up data to researchers, which is the focus of the 2019 paper in the *Journal of Medical Internet Research* [3]:

From early on, researchers came to Crisis Text Line asking for access to data. This prompted even more reflection. We had significant data and we were seeing trends that had significant implications for far more than our service. (...) This then led to the more complicated issue of whether or not to allow external researchers to study our data with an eye towards scholarship. (...) Our texters come to us in their darkest hours. Our data was opening up internal questions right and left about how to best support them. We don’t have the internal resources to analyze the data to answer all of our questions, to improve our knowledge base in ways that can help texters. I knew that having additional help from researchers could help us learn in ways that would improve training of counselors and help people down the line. I also knew that what we were learning internally might be useful to other service providers in the mental health space and I felt queasy that we were not sharing what we had learned to help others.

The 2019 paper deals with the question of how these textual data could be ethically shared with academic researchers to answer a variety of research questions. As Pisani and colleagues observed, “few companies are willing to take on the potential work and risks involved in noncommercial data sharing, and the scientific and societal potential of their data goes unrealized” [1], so to Crisis Text Line’s credit the nonprofit organization applied for a grant from the Robert Wood Johnson Foundation to create a pilot program for data sharing with academic researchers. The paper described “the process of defining core challenges underlying data sharing in technology-academia partnerships; discusses Crisis Text Line’s trial solutions to these challenges; and offers lessons learned that might inform other technology companies’ data-sharing partnerships” [1]. This is a viewpoint paper, not an original paper, and the paper in the *Journal of Medical Internet Research* does not use any of the data collected by Crisis Text Line nor does it share the results of the subprojects that Crisis Text Line enabled with their data-sharing program, rather it deals with the meta-question of how such data can be ethically shared with researchers. To that end, Crisis Text Line assembled a data ethics committee consisting of external academic researchers and technology experts (many of whom are coauthors of the paper), convened by then Crisis Text Line’s chief data scientist and Crisis Text Line cofounder, Bob Filbin, who is also a coauthor on the paper. The ethics committee advised Crisis Text Line on processes and assessed the research proposals of other researchers who applied for access to Crisis Text Line’s data. The paper proposes some general guidelines for other organizations that want to share data with third parties in a *noncommercial* context. The paper is seen by many as an important contribution to the complex question of how organizations can ethically share data with academics while preserving the privacy and integrity of the data, as evidenced by the selection of the article as “best paper of 2019” for the 2020 International Medical Informatics Association (IMIA) Yearbook, Special Section on Ethics in Health Informatics [4].

## Data Ethics Called in Question

In November 2021, Tim Reierson, a former volunteer for the Crisis Text Line, and an advocate seeking reform of data ethics at Crisis Text Line and 988 Lifeline, published a 13-page open letter in response to the article published in the *Journal of Medical Internet Research*, where he raised several concerns related to informed consent and an alleged conflict of interest [5]; the full document was sent to us in February 2022.

Reierson’s core concern related to *informed consent* was that the paper described the process where Crisis Text Line provided “texters with a link to an easy-to-understand Terms of Service” (Table 1 in [1]), thereby—according to Reierson—“establishing a Terms of Service consent standard.” While we as *Journal of Medical Internet Research* editors agree that offering a terms of service (ToS) link does not necessarily equate to informed consent, we do not agree with the interpretation that this viewpoint paper establishes this as a generalizable “informed consent standard.” Rather, the guidelines proposed in the viewpoint (Table 1 in [1]) include that researchers “inform users in an unobtrusive way that anonymized data are shared with select research partners”, which was implemented by Crisis Text Line by referring users to their ToS. But the guidelines proposed in the viewpoint paper also had other critical requirements. Notably, they also require that academics who use (anonymized) Crisis Text Line data for research obtain institutional review board (IRB) approval for their data analysis projects (Table 1 in [1]: “Establish a review process that includes outside academics and ethics experts”).

Informed consent is ideal but not always possible, especially on the internet [6]. There are some circumstances, for example, in urgent or emergency care settings, or in public health practice, where informed consent is considered legally effective when considering contextual variables [7]. Additionally, secondary use of data that has been de-identified or anonymized (as was the case with Crisis Text Line data) is usually allowable in the absence of explicit consent from the data subject [8]. Thus, the critique that the Crisis Text Line clientele can not be expected to read the terms of use is valid, but it is ultimately up to the IRBs to assess the risk—which is a key component mentioned in the Pisani paper [1].

Regarding commercial use, Reierson in his letter to JMIR Publications pointed to the fact that Crisis Text Line had also launched a for-profit subsidiary, Loris.ai, to which Crisis Text Line data was licensed in order to train artificial intelligence (AI) to handle text exchanges for customer service. These events took place after the study period.

Less than 2 months after Reierson made the letter to JMIR Publications public (but before we had seen it), on January 28, 2022, Politico ran a story bringing public scrutiny to the fact that Crisis Text Line had created Loris.ai as a profit-generating entity [9], which was met with widespread public “anger and disgust” [3], and prompted a letter from US Federal Communications Commission’s commissioner, Brendan Carr, to Crisis Text Line and Loris.ai demanding they stop this practice immediately [10]. Three days later, Crisis Text Line backed down and “ended the data-sharing relationship with Loris” and requested “that Loris delete the data it has received from Crisis Text Line” [11,12].

## Publication Ethics and Corrigendum of the Conflicts of Interest Section

Reierson’s concern regarding the *conflict of interest* was that “Crisis Text Line itself had a vested monetary interest in commercial use of the data through its’ subsidiary Loris.ai, and therefore vested interest in the 2019 paper’s finding [*sic*] that the crisis conversation data is ethically sourced for research purposes.” Here, it is important to note that from our point of view as *Journal of Medical Internet Research* editors, the term “finding” is slightly misleading because this is not an empirical study, rather it is a viewpoint paper, largely written by members of the independent Data Ethics Committee consisting of independent and respected ethicists and academics, describing how Crisis Text Line handled data sharing for research and outlining ethical challenges and how they were addressed by the Crisis Text Line’s Data Ethics Committee.

From a publication ethics perspective, we are less concerned about the ethics of the arrangement between Crisis Text Line and Loris.ai, but primarily about whether any of the individual contributing authors benefitted (or had the potential to benefit) financially from Loris.ai or had any other ties to Loris.ai, as this would arguably be something that should have been disclosed to the editor and reviewers on submission of the paper [13]. Even though the 2019 paper published in the *Journal of Medical Internet Research* was about *noncommercial* data sharing for academic research, any hypothetical ties between authors and a company that benefits from Crisis Text Line data-sharing guidelines could have influenced the viewpoint.

We shared Tim Reiersons’ letter with the corresponding author and received a reply on March 30, 2022, reassuring us that the academic members of the Data Ethics Committee were uncompensated and that there were no further conflicts of interest to disclose. We had no evidence of any wrongdoing that would require a retraction or even issuing an editorial expression of concern. Still, we wanted readers to be aware of the debate (in particular around informed consent) and invited Tim Reierson to submit a condensed commentary for publication alongside the article [14], and we also asked the original authors to respond [15]. We also decided to publish a corrigendum [16] to clarify author relationships with the noncommercial and commercial entities in greater detail [16].

## Informed Consent in the Age of AI and Machine Learning

The invited commentary by Reierson highlights complex concerns regarding consent for research, data sharing, and machine learning [14], which are of course amplified given the initial intent of Crisis Text Line to share data with a *commercial* entity, Loris.ai.

Tim Reierson asks legitimate questions on whether it is sufficient to have users in crisis accept a ToS document (which in all likelihood nobody reads). Even former board chair of Crisis Text Line, Danah Boyd, readily admitted that a ToS is not consent [3]:

I knew how little data exists in the mental health space, how much we had tried to learn from others, how beneficial knowledge could be to others working in the mental health ecosystem. I also knew that people who came to us in crisis were not consenting to be studied. Yes, there was a terms of service that could contractually permit such use, but I knew darn straight that no one would read it, and advised everyone involved to proceed as such.

Informed consent is a basic tenet for research; on the other hand, it can be waived when data are deidentified. IRBs routinely grant exemptions to the informed consent ideal when data are deidentified. While such assessments can be made in an academic context, many companies do not have IRBs that could assess the risk or ethical implications if data are analyzed, manually or with machine learning, and the reidentification risks are often not known.

The question of what the role of informed consent plays when academics or companies use data generated on the internet is one of the most vexing questions of our time and a topic of ongoing debate. It is also precisely why we published the 2019 viewpoint paper by Pisani et al [1], which illustrates in an exemplary way how organizations can and should deal with this risk. Ideally, organizations (profit or nonprofit) assemble independent ethics boards, like Crisis Text Line did. Independence is key—the ethics board should consist of independent academics who do not have a stake in the success of the business or organization.

As Danah Boyd further writes, “There have been heated debates in my field about whether or not it is ethical to use corporate trace data without the consent of users to advance scientific knowledge” [3]. Proponents of waiving informed consent requirements for analyzing anonymized big data argue that users implicitly consent to data mining and research by using “free” services—one could argue that people accept this bargain as a trade-off to receiving free services and free information. In the age of generative AI—where large parts of the internet, including discussion boards and other venues that once may have been deemed “private,” are scoured by AI bots—users already may have adjusted their expectations regarding privacy and anonymity.

However, when it comes to digital health data, there is an important distinction to be made between noncommercial and commercial use of the data, and perhaps this was the cardinal mistake made by Crisis Text Line—to first communicate that data will never be shared with commercial entities, and then change course, assuming that people who may be fine with giving data (even anonymized) for research purposes to a noncommercial entity (including the volunteers at Crisis Text Line) are also OK with having these anonymized data shared with a commercial entity for machine learning purposes. Research in this journal [17] has shown that people generally distrust data use in a commercial context (technology companies, pharmaceutical companies) more than data sharing for pure research purposes.

The debate over informed consent in the use of “deidentified” internet-generated data for research and commercial use is complex, multifaceted, and constantly evolving.

In a recent 2024 paper [18], the authors argue that especially for digital mental health and other vulnerable populations, the standard to deidentify data to waive informed consent may not be sufficient, and other criteria should be used to address social justice issues. To mitigate the “social risk” of using deidentified data for research without explicit permission from participants, the authors urge researchers to consider the following additional guidelines [18]:

- create socially valuable knowledge,
- fairly share the benefits and burdens of research,
- be transparent about data use,
- create mechanisms for withdrawal of data,
- ensure that stakeholders can provide input into the design and implementation of the research, and
- responsibly report results.

Balancing these ethical considerations with the practical needs of research and innovation is a challenge that requires ongoing dialogue and thoughtful regulation. Ultimately, finding a middle ground that protects individuals’ rights while fostering scientific and technological advancement is crucial for navigating this complex issue.

## Conclusion

In summary, the commentary of Reierson [14] and the response of Pisani et al [15] highlight the difficult ethical questions nonprofit organizations and companies face when obtaining, analyzing, and sharing data with academics, and even more so for commercial use. The paper by Pisani et al [1] is commendable in that it lays open how data sharing for research has been handled by a nonprofit organization, and the approach of assembling an external data ethics committee to help vet research proposals from other researchers is exemplary. Unfortunately, this has been somewhat tainted by events that occurred after we published the paper, when it emerged that Crisis Text Line made the controversial decision to also share the data with its commercial subsidiary for machine learning purposes. The Data Ethics Committee members who authored the 2019 *Journal of Medical Internet Research* paper were apparently not involved or consulted on that. We see the controversy around Loris.ai as a separate debate that does not invalidate the guidelines and approach for noncommercial data sharing published in the 2019 paper. Many organizations (including nonprofits, hospitals, and research centers) have business development officers and commercialization units that think about how to harness the data they have to advance knowledge and how to commercialize their data in an ethical manner. As such, in the era of generative AI and machine learning, nonprofits creating spin-offs or holding equity in companies that commercialize deidentified data may become increasingly common, and the Crisis Text Line and Loris.ai case may serve as a cautionary lesson on the negative public perception of such arrangements.

## Abbreviations

AI: artificial intelligence
CEO: chief executive officer
IMIA: International Medical Informatics Association
IRB: institutional review board
ToS: terms of service
